# Left-right asymmetry and attractor-like dynamics of dog’s tail wagging during dog-human interactions

**DOI:** 10.1016/j.isci.2022.104747

**Published:** 2022-07-09

**Authors:** Wei Ren, Pengfei Wei, Shan Yu, Yong Q. Zhang

**Affiliations:** 1 State Key Laboratory for Molecular Developmental Biology, Institute of Genetics and Developmental Biology, Chinese Academy of Sciences, Beijing 100101, China; 2 College of Life Sciences, University of Chinese Academy of Sciences, Beijing 100049, China; 3 Shenzhen Key Lab of Neuropsychiatric Modulation and Collaborative Innovation Center for Brain Science, Guangdong Provincial Key Laboratory of Brain Connectome and Behavior, Shenzhen Institutes of Advanced Technology, Chinese Academy of Sciences, Shenzhen 518055, China; 4 Shenzhen-Hong Kong Institute of Brain Science-Shenzhen Fundamental Research Institutions, Shenzhen 518055, China; 5 Brainnetome Center and National Laboratory of Pattern Recognition, Institute of Automation, Chinese Academy of Sciences, Beijing 100190, China

**Keywords:** Social interaction, Canine behavior, Neuroscience

## Abstract

Tail wagging plays an important role in social interactions, e.g., dogs show asymmetrical tail wagging in response to different social stimuli. However, the effects of social cues on tail wagging and the intrinsic organization of wagging behavior remain largely unknown. Here, we developed a platform using a deep-learning-based motion-tracking technique to extract and analyze the movement trajectory of a dog’s tail tip during dog-human interactions. Individual dogs exhibited unique and stable wagging characteristics. We further found that tail wagging developed asymmetry toward the right side over three days of dog-human interactions, suggesting that it is a time-sensitive indicator of social familiarity. In addition, wagging appeared to follow an attractor-like dynamic process consisting of stable states and unstable, transitional states. Together, these results revealed sophisticated characteristics and organization of a dog’s tail-wagging behavior during interactions with humans, providing a useful paradigm for studying dogs’ social behaviors and the underlying neural mechanisms.

## Highlights

• Tail-wagging kinematics was analyzed by AI-based method with high resolution
• Tail wagging shifts from left side to right side as dogs familiarize with humans
• Individual dogs show a distinct spatiotemporal pattern of tail wagging
• Lyapunov exponent-based analysis reveals attractor-like dynamics in tail wagging

## Graphical abstract

**Figure:**
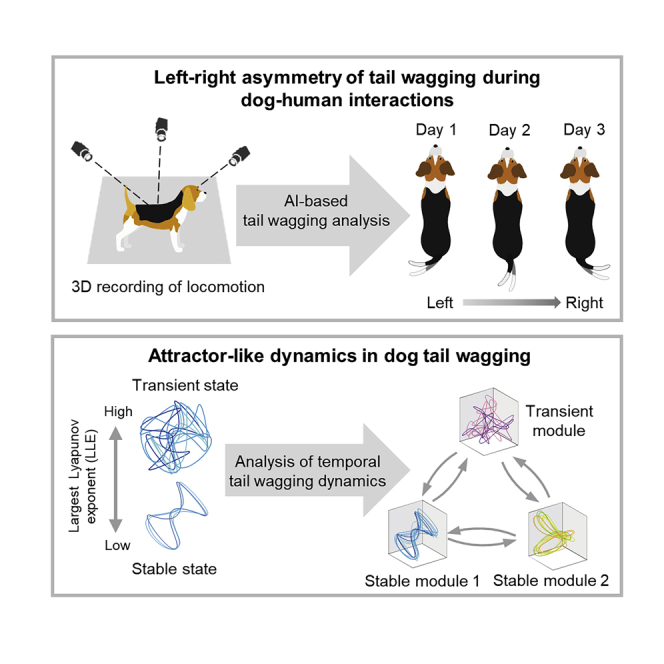

## Introduction

Social behaviors are important in understanding how individual animals interact with each other (Chen and Hong, 2018). Although social behaviors typically involve within-species interactions, they may also be directed toward individuals of other species (Carter and Porges, 2016). Tail wagging is an important example of such behavior in dog-dog and dog-human interactions.

Affiliative relationships developed between humans and dogs provide a unique model for investigating social relationships between species. Previous studies revealed that tail wagging was associated with a dog’s inner state, related to the emotional state in humans, and conveyed sophisticated information during social interactions (Bradshaw and Nott, 1995; Goodwin et al., 1997; Ortolani, 1999; Quaranta et al., 2007; Wansbrough, 1996). Tail wagging is thus a context-specific behavior that reflects the inner state of the dog, such as its friendliness, confidence, anxiety, and aggressiveness toward threats (Serpell, 1995). For example, higher tail positions are associated with confidence and/or aggression, while lowered tail positions could be a neutral signal or reflect fear and/or submission (Kleiman, 1972; Tembrock, 1968). In addition, one study reported more pronounced asymmetric tail wagging toward the right side of the dog’s body axis when facing the dog’s owner, compared with a stranger (Quaranta et al., 2007). A follow-up study by the same groups further showed that this wagging asymmetry can be detected by other dogs (Siniscalchi et al., 2013). However, traditional video-based manual analyses of tail wagging in dogs are usually time-consuming and limited in terms of capacity and accuracy, thus preventing an in-depth understanding of dog tail-wagging behavior.

In recent years, there has been rapid technical progress in automatic pose estimation (Graving et al., 2019; Mathis et al., 2018; Pereira et al., 2019), 3D reconstruction (Bala et al., 2020; Dunn et al., 2021; Nath et al., 2019), and behavior identification (Hsu and Yttri, 2021; Huang et al., 2021, 2022; Liu et al., 2022; Marshall et al., 2021; Zanon et al., 2021), providing new tools to quantify animal behaviors for various species and in different conditions. To better characterize tail wagging, we integrated deep-learning-based pose estimation, as well as 3D reconstruction to track the tail-tip trajectory of free-moving Beagle dogs with a high spatiotemporal resolution, and analyzed the wagging dynamics during dog-human interactions. Our data highlight the power of artificial-intelligence-based approaches for revealing previously undetectable features of tail wagging, including distinct wagging characteristics for individual animals, social interaction-induced shifts in left-right asymmetry, and attractor-like dynamics of tail wagging.

## Results and discussion

### Development of a platform for high spatiotemporal resolution analysis of tail-wagging kinematics

**Figure 1:**
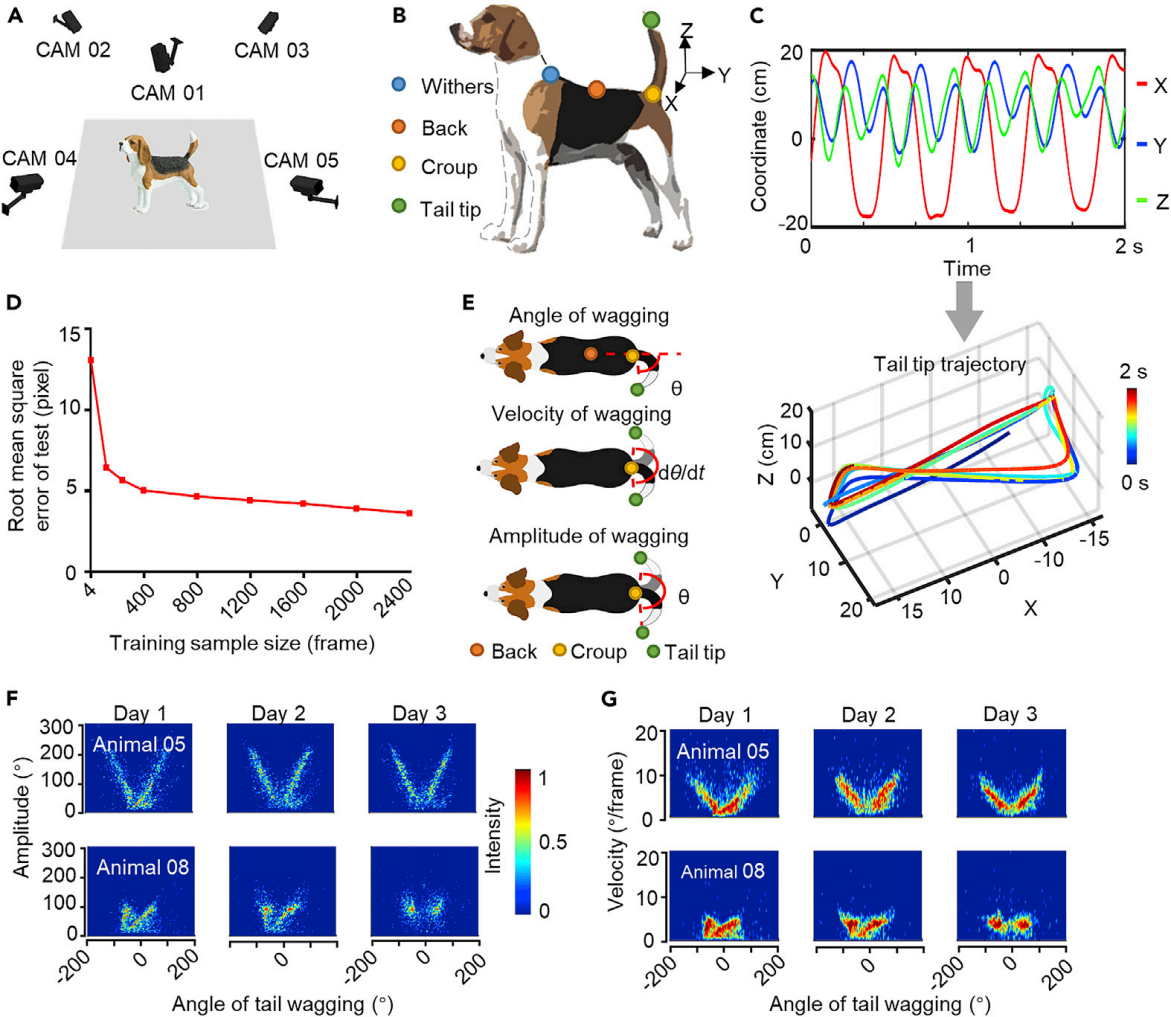
Tracking of dog tail wagging and analysis of kinematic parameters of wagging (A). Tail wagging was recorded simultaneously using five cameras placed at the four corners and the center above a rectangular arena. (B). Four tracking points (color-coded) used for capturing the tail-tip trajectory, estimated using a pre-trained deep neural network in DLC. (C). Example of tail-tip trajectory for 2 s. Trajectories corresponding to the *x*, *y*, and *z* dimensions (top) and reconstructed 3D trajectory (bottom). The lapse of time from the starting point of the trajectory is color-coded. (D). Root mean square error (RMSE) averaged for all tracking points in the testing frames plotted as a function of the training sample size used to train the neural network. (E). Schematic of three kinematic parameters used to characterize tail wagging, including angle (top), velocity (middle), and amplitude of wagging (bottom). Parameters estimated based on position and movements of three tracking points at the back, croup, and tail tip (color-coded). Horizontal red dashed line indicates spine axis connecting the back and croup. (F and G). Joint distributions of angle vs. amplitude (G) and velocity vs. angle (H) of tail wagging for two animals (top row: Animal 5, bottom row: Animal 8) over 3 consecutive days. Joint distribution intensity (color-coded) normalized from 0 to 1.

To characterize the detailed kinematic pattern of dog tail wagging, we developed a 3D motion-tracking system (Figure 1A) based on recent advances in video-based pose-estimation techniques such as DeepLabCut (Mathis et al., 2018) and 3D reconstruction (Schonberger and Frahm, 2016). The system integrated high-speed cameras (150 frames/s) and a computer-controlled image acquisition module, allowing us to locate the 3D position of the withers, back, croup, and tail tip (Figure 1B) in free-moving dogs. We recorded tail wagging during dog-human interactions for one 5-min session per day for 3 consecutive days. During each session, the experimenter kept a neutral posture and provided treats to the dog without direct contact. The deep neural network-based model DeepLabCut (DLC) (Mathis et al., 2018) was trained to locate the four body landmarks in 2D for each recorded frame, and location data from all five cameras were combined to calculate the position of the body landmarks in 3D (Figure 1C). Unlike previous studies, in which the positions of the tail were judged manually at intervals of a few seconds (Quaranta et al., 2007), the current system was able to extract details of tail movements with a temporal resolution < 10 ms. To examine the accuracy of the automatically identified body-part locations, we manually labeled the body landmark positions in 3000 frames selected uniformly from all the videos, and then split the data randomly into a training set and a test set containing 2400 and 600 frames, respectively. When the number of labeled frames in the training set reached 2400, the root mean square error (RMSE) achieved in the test set approximated the human-level accuracy (< 5 pixels), as previously defined (Mathis et al., 2018) (Figure 1D). This well-trained deep neural network was then used to generate position data for all recordings for further analyses.

To characterize tail-wagging kinematics, we analyzed three parameters: the angle (maximum extent of tail wagging on both sides, ranging from −180° (left) to 180° (right) relative to the body axis), amplitude (absolute difference in adjacent angle, as defined above, of tail wagging, ranging from 0° to 360°), and velocity (angular velocity averaged within a wagging bout, which is defined as a minimum segment of wagging from the most-left to the most-right side and back again, ranging from 0°/frame to 16°/frame) of tail wagging (Figure 1E). Figures 1F and 1G show examples of the joint distribution of angle vs. amplitude and angle vs. velocity, respectively, for two animals across 3 days. We found that individual animals exhibited unique joint distributions that were stable across days, suggesting individualized tail-wagging kinematic characteristics in dogs.

### Tail wagging shifts from left side to right side as dogs familiarize with humans

**Figure 2:**
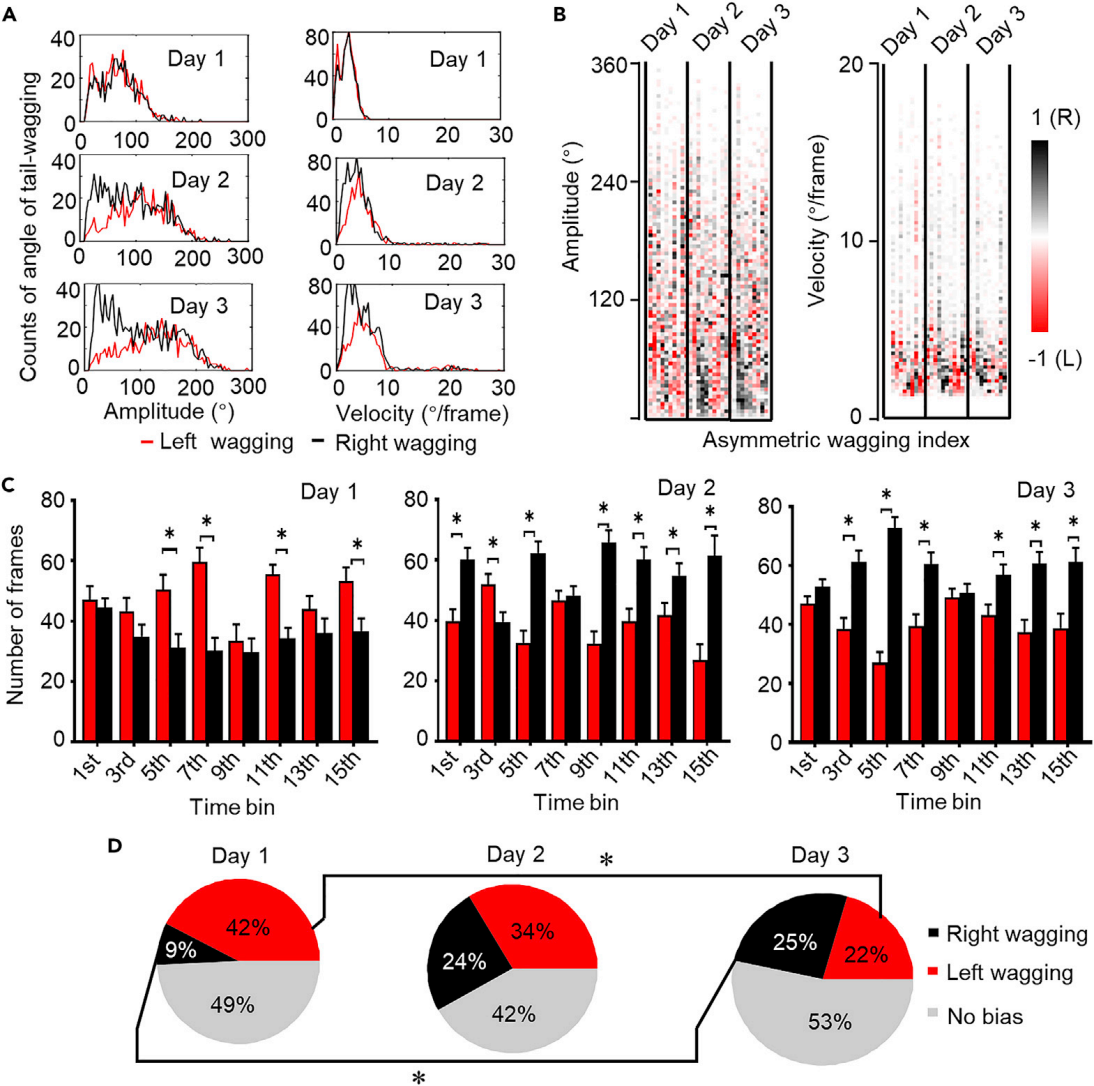
Tail wagging at low amplitude and velocity shifts from left- to right-side bias during dog-human interactions (A). Counts of maximum angles of left-sided and right-sided tail wagging (color-coded) plotted as a function of amplitude (left panels) and velocity (right panels) over 3 days of Animal 4. (B). Asymmetric tail-wagging index (−1/1 indicates complete left/right bias) for all 10 animals versus amplitude (left panel) and velocity (right panel) over 3 days. (C). Numbers of frames including tail wagging to the left and right side (color-coded) plotted for 3 consecutive days of Animal 4. For each day, the 5-min recording session was divided into 15 segments, each of which was further divided into 30 sub-segments. For visual clarity, the mean and standard error are shown for every other segment. ∗p < 0.05 (Welch’s *t*-test). (D). Pooled results of fractions of segments showing left, right, and no bias (color-coded) for all 10 animals in 3 days. ∗p < 0.05 (Mann-Whitney test).

We noted that the joint distributions were asymmetric in terms of the angles of tail wagging (Figures 1F and 1G). We therefore quantified the amplitude and velocity of tail wagging as a function of the angle, with left-sided and right-sided wagging analyzed separately. Representative results for Animal 4 are shown in Figure 2A. This dog exhibited largely left-right symmetric wagging on the first day, and subsequently developed bias toward the right side in the following days, especially in the subset of wagging bouts with medium to low amplitudes (< 100°) and velocities (< 6°/frame) (Figures 2A and 2B). We further introduced the asymmetric wagging index to quantify the extent of asymmetry in all 10 animals, with −1 indicating complete bias toward the left while 1 indicating complete bias toward the right. The results showed that asymmetric tail wagging toward the right side developed gradually over 3 days (Figure 2B).

To quantify the time-dependent development of wagging asymmetry, we calculated the proportion of left- and right-sided wagging during each day’s 5-min interaction session, divided evenly into 15 time windows, each lasting 20 s. The bias toward left- and right-sided wagging was analyzed by Welch’s *t-*test for individual animals (Figure 2C for animal 4). The fraction of left-wagging for all animals decreased significantly from 42% on day 1 to 22% on day 3 and the fraction of right-wagging increased significantly from 9% to 25% (Figure 2D). There was no significant difference between the second day and any of the other 2 days.

To see if the bias toward right-sided wagging persists or changes over a longer time, we extended the analysis of wagging bias for 2 more days after the original 3-day social interaction in three additional dogs. In these animals, the proportion of left-wagging decreased significantly on the 3^rd^, 4^th^, and 5^th^ days compared with the 1^st^ day, while the fraction of right-wagging increased significantly on the 2^nd^, 3^rd^, 4^th^, and 5^th^ days compared with the 1^st^ day. There were no significant differences among the 3^rd^, 4^th^, and 5^th^ days (see Figure S1).

As a control for the left-right asymmetry of tail-wagging angles shown above, we compared the frequency, amplitude, and velocity of wagging for ten animals across 3 days (see Figure S2). Although there were statistically significant changes in the three parameters across three days, the changes were either too small (< 6% for frequency, < 5% for amplitude, and < 7% for velocity) or not consistent within three days, highlighting the consistent and pronounced changes in the left-right asymmetry of wagging angles as a unique phenomenon during dog-human interactions.

Left-right asymmetries in brain function and behavior are widespread in both humans and non-human species (Rogers et al., 2013). For example, Broca’s area and Wernicke’s area, associated with the production of speech and comprehension of speech, respectively, are located in the left hemisphere for about 95% of right-handers but about 70% of left-handers (Griggs, 2010). A previous study found that pet dogs exhibited right-sided bias in tail wagging, which was more pronounced when the dog interacted with its owner than with an unfamiliar human (Quaranta et al., 2007). In the present study, we showed that laboratory Beagles exhibited left-sided bias or no bias in tail wagging when faced with the unfamiliar experimenter on the first day, but the left-sided bias significantly decreased and converted to right-sided bias within 3 days of dog-human interactions. Currently, the neural mechanisms underlying this asymmetric tail wagging are unclear. A previous study suggested that asymmetric tail wagging might be related to brain hemisphere-specific emotional processing (Quaranta et al., 2007). Positive and negative emotional states have been associated with left- and right-sided activation of the prefrontal cortex in humans (Ahern and Schwartz, 1985; Davidson, 1996, 1998; Davidson et al., 1990; Jones and Fox, 1992). Given that the prefrontal cortex is involved in the cognitive control of motor activities (Goldman-Rakic, 1987), it is conceivable that emotion-dependent asymmetric activation may be manifested at the behavioral level. The observed asymmetric tail wagging might reflect changes in the emotional state of the dogs during their interactions with humans. Our results with laboratory Beagles and previously reported asymmetric tail wagging in pet dogs indicate that the left- and right-wagging bias might be indicators of negative and positive effects, respectively. Compared with laboratory dogs, pet dogs interact with humans more frequently and the valence of interaction is probably more pronounced, which may explain why they exhibited a right-sided bias (positive affect) in tail wagging even when faced with an unfamiliar human, while laboratory dogs exhibited a left-sided bias (negative affect) when faced with a stranger. Further studies are needed to verify this hypothesis. Nevertheless, we found that the tail-wagging bias could shift from left to right with just a few 5-min sessions of dog-human interactions (one 5-min session per day for 3 consecutive days), suggesting that tail wagging may be an easily recognizable and time-sensitive indicator of a dog’s social-related inner state.

### Individual dogs show a distinct spatiotemporal pattern of tail wagging

**Figure 3:**
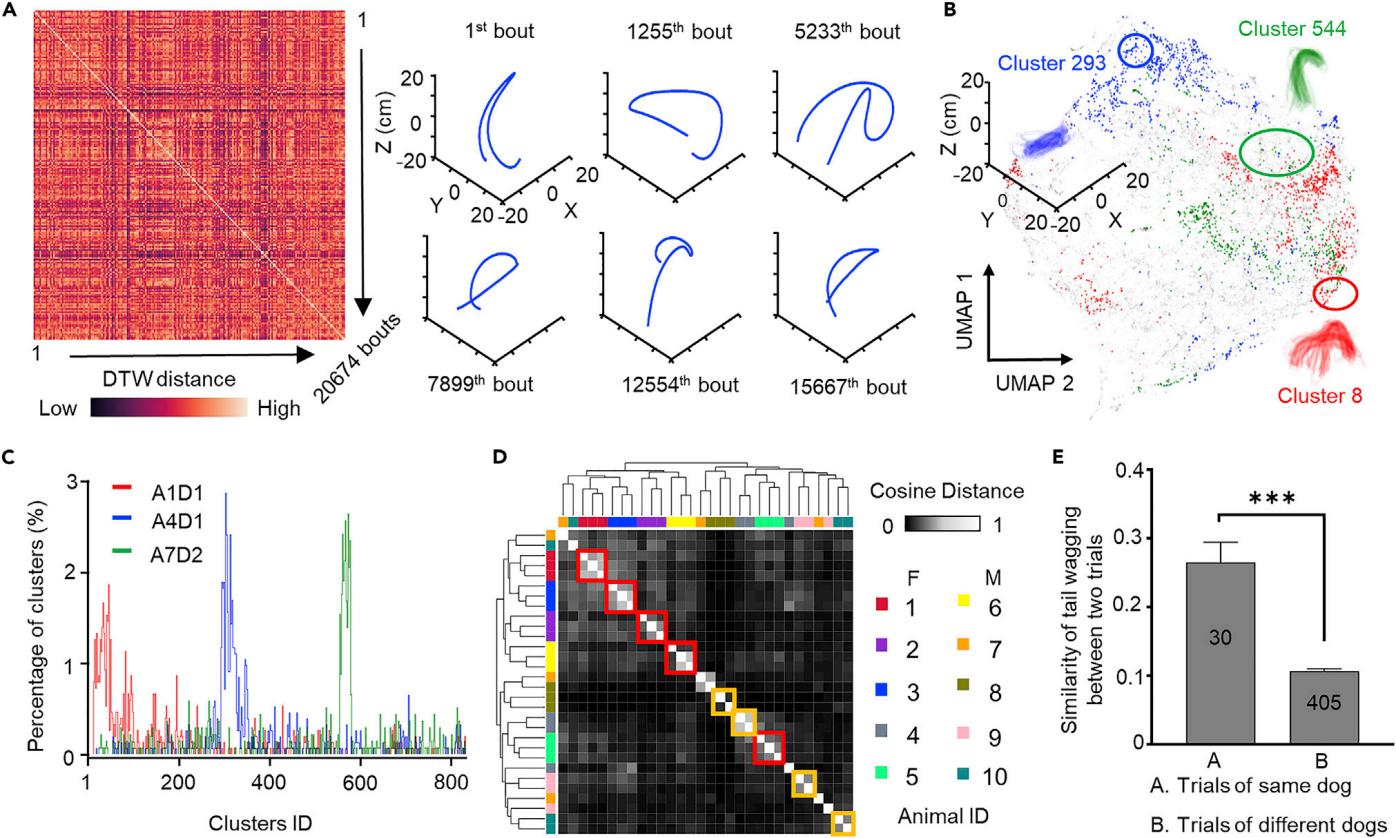
Unsupervised clustering reveals conserved tail wagging in individual dogs (A). Dynamic time warping (DTW) distance (color-coded) between different bouts in all dogs shown in a similarity matrix. Six representative bouts are shown on the right. (B). Clustering results of wagging bouts recorded in three sessions from three different animals. The results are embedded by uniform manifold approximation and projection (UMAP) into a 2D plane (UMAP1 and UMAP2). Dots represent individual bouts; colors indicate clusters to which the dots belong. Representative clusters for three animals (color-coded) are shown in blow-ups. (C). Cluster ID distribution from three sessions (A: animal; D: day; color-coded) is plotted. (D). Matrix of similarity distances (color-coded) between pairs of bouts, analyzed by hierarchical clustering. Three sessions for the same dog clustered together are highlighted by red rectangles, and two sessions for the same dog clustered together are highlighted by orange rectangles. F: females, M: males. All animal IDs are color-coded. (E). Similarity of tail wagging between sessions for the same and different dogs, calculated based on cosine distance. The sample size was noted in the bar. ∗∗∗p < 0.001 by Welch’s *t*-test.

In addition to wagging asymmetry, we also noted that individual dogs exhibited robust wagging characteristics (Figures 1F and 1G). We examined if this phenomenon was due to a stereotyped wagging pattern for each animal by analyzing the movement trajectories of 20,674 wagging bouts in 10 animals. We first calculated the similarity between each pair of bouts using dynamic time warping (DTW) (Figure 3A). We then clustered the bouts using affinity propagation (AP) clustering (Frey and Dueck, 2007), resulting in 851 clusters of wagging bouts for all 30 dog-human interaction sessions for the 10 animals. The silhouette value estimates how similar an object is to its own cluster when compared to other clusters. The silhouette has a value between −1 and +1, with a high value indicating that the object is well matched to its own cluster but poorly matched to nearby clusters (Rousseeuw, 1987). By computing the silhouette score for each wagging bout, we assessed the quality of the clustering. The percentage of silhouette scores that were higher than 0 was 68.5%. We analyzed the frequency of occurrence of each cluster in each session, and presented the cluster distributions for each 5-min session to represent tail-wagging patterns (Figure 3B). We then performed uniform manifold approximation and projection (UMAP) embedding using the Euclidean distances between bouts (Figure 3C), which showed that each animal tended to have distinct clusters of wagging. Finally, we calculated the similarity defined by the cosine distance between pairs of sessions, which produced a similarity matrix by hierarchical clustering (Figure 3D). We found that all three sessions for the same dog were grouped in the same cluster for five of the ten dogs, while two sessions for the same dog were grouped in the same cluster for other four dogs (Figure 3D). Notably, all three sessions for the same dog were grouped in the same cluster for four of the five females compared with only one of the five males (Figure 3D), suggesting that spatiotemporal patterns of tail wagging may be more stable in females than males. Further analysis by Welch’s *t*-test showed that tail wagging was significantly more similar across different sessions for the same dog than between different dogs (∗∗∗p < 0.001) (Figure 3E). These results, together with the kinematics analysis (Figure 1), showed that tail wagging in dogs exhibited unique and stable features for individual animals, similar to the distinct gaits of individual humans (Johansson, 1973; Stevenage et al., 1999).

### Attractor-like dynamics in dog tail wagging

**Figure 4:**
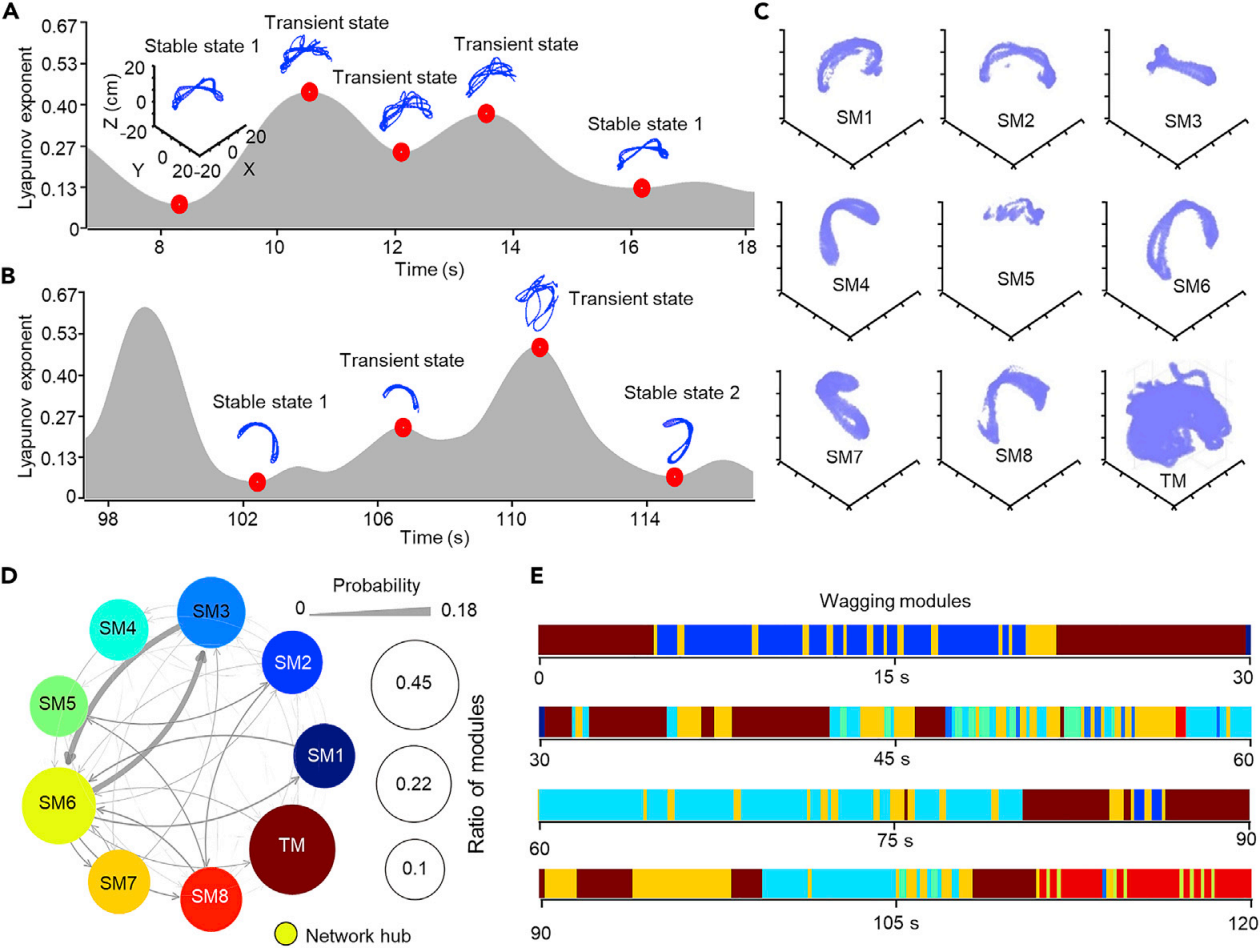
Temporal organization of tail wagging during dog-human interaction (A and B). Two representative episodes of LLE evolution for wagging behaviors recorded in session 2 of Animal 9. Local extremes of LLE are highlighted by red dots and corresponding wagging trajectories are shown above the dots. A stable state with low LLE may transit back to itself through a transient state with high LLE (A) or transit to another stable state (B). (C). All nine tail-wagging modules identified for session 2 of Animal 9. SM: stable module. TM: transient module. (D). Transitions between tail-wagging modules for session 2 of Animal 9, illustrated by weighted graph. Different wagging modules represented by color-coded vertex of the graph. Transition probabilities between different modules represented by weighted lines (ranging from 0 to 0.18). The ratio (0.1–0.45) of each module is represented by the size of the circle. The network hub SM6 is noted. (E). Ethogram of wagging modules during the first 120 s of dog-human social interaction, divided into four 30-s epochs from session 2 of Animal 9.

We then examined the temporal organization of wagging behaviors. We hypothesized that tail wagging, similar to other behaviors, might be composed of different modules, each with highly stereotypical patterns. To test this hypothesis, we analyzed the detailed wagging patterns in all 10 animals. We first calculated the largest Lyapunov exponent (LLE, ranging from −0.01 to 0.98) of the tail-wagging trajectory, which quantified the divergence and stability of the trajectory. A threshold of LLE = 0.18 (25^th^ percentile of LLE distribution) was used to divide the segments of tail-wagging trajectory into two categories: stable state (LLE ≤ 0.18), in which the trajectory showed stereotyped patterns, and unstable states (LLE > 0.18), in which no stereotyped moving pattern could be found. As shown in Figure 4 for animal 9, a stable state may transit back to itself through an unstable state (Figure 4A) or to another stable state (Figure 4B).

To further categorize these states, we examined the overall organization of tail wagging by clustering stable wagging segments (LLE < 0.18) of individual sessions using the DTW-AP clustering which generated a large number of micro-modules of wagging patterns, and the percentage of silhouette scores that were higher than 0 was 90%. To provide a simpler picture of the overall modular organization, the representative segments, defined as the segments closest to the center of individual micro-modules, were then subjected to further DTW-AP clustering resulting in mini-modules. Finally, the representative segments of mini-modules were subjected to *k*-means clustering to group similar mini-modules into eight stable modules (SM) of tail wagging (Figure 4C). The wagging within unstable wagging segments was grouped as a transient module (TM). These three-step clustering processes by DTW-AP (two rounds) and *k*-means revealed that wagging in individual animals involved a dynamic process of transitions among different modules (Figures 4D and 4E). The transitions among different modules including SM and TM are illustrated by a graph with weighted connections indicating different transition probabilities (Figure 4D). Figure 4E shows an example of temporal transitions of different modules within a 120-s segment of dog-human social interaction.

We thus revealed that dogs’ tail wagging comprised stereotyped moving patterns as modules. These results suggest that the neural population activity controlling the tail movement may consist of attractors, which are self-maintained stable states discovered in a wide variety of dynamic systems, including neural networks *in vivo*. For example, previous studies have found ring attractor dynamics in the *Drosophila* brain (Kim et al., 2017), discrete attractor dynamics in the mouse anterior lateral motor cortex (Inagaki et al., 2019) and head-direction circuit (Chaudhuri et al., 2019), and attractor-like oscillatory activities in the motor cortex of monkeys performing hand-reaching behavior (Churchland et al., 2012). As tail-wagging behavior can be viewed as an easily accessible readout of the motor control circuit in dogs, further studies to analyze both neural population activity and tail wagging simultaneously in dogs would thus help us to understand if and how attractor-like neural dynamics produce behaviors consisting of distinct modules. Moreover, the dog represents an innovative and unique animal model for a range of human neuropsychiatric disorders, including autism spectrum disorder (Bunford et al., 2017). We believe that the present work will provide useful paradigms and tools for studying dogs’ social behaviors and their anomalies in corresponding disease models.

### Limitations of the study

In the present study, we uncovered previously unappreciated kinematics of dog tail wagging by a deep-learning-based analysis. Still, there are a few limitations of our study. First, the present study focuses on computational analysis of wagging behaviors. Integrating quantitative analyses of animal behaviors with neural recordings will reveal the neuronal correlates of the behavioral traits we described here. Also, for behavior analyses, future work would benefit from a multi-modal approach that can monitor not only tail wagging but also gaze, facial expressions, etc., to have a more comprehensive measure of dog’s social behaviors. Second, in the present, we studied tail wagging in normal laboratory Beagles. To fulfill the potential of the paradigm we developed, it would be important to apply it to dog models of psychiatric diseases such as depression and autism (Bunford et al., 2017). Finally, it would be interesting to characterize tail wagging at different developmental stages of dogs. Socialization at 3 to 14 weeks of age prepares a dog to be comfortable with other animals, people, and environments (Seksel, 1997). Analyzing tail wagging before, during, and after the socialization period by the platform we presented here may provide new insights into how the wagging patterns are developed in social developmental contexts.

## STAR★Methods

### Key resources table

**Table:** 

REAGENT or RESOURCE	SOURCE	IDENTIFIER
**Deposited data**		

Processed behavioral data	This paper	

**Experimental models: Organisms/strains**		

Beagle	Beijing Sinogene Biotechnology Co. Ltd	N/A

**Software and algorithms**		

Matlab	www.mathworks.com	RRID:SCR_001622
GraphPad Prism 8	www.graphpad.com	RRID:SCR_002798
Python 2.7	www.python.org	RRID:SCR_008394
Python 3.4	www.python.org	RRID:SCR_008394

### Resource availability

#### Lead contact

Further information and requests for resources should be directed to and will be fulfilled by the lead contact, Yong Q. Zhang (yqzhang@genetics.ac.cn).

#### Materials availability

This study did not generate any new reagents.

### Experimental model and subject details

All animal-related protocols were approved in advance by The Animal Care and Use Committee of the Institute of Genetics and Developmental Biology (AP2022001). Ten Beagle dogs (1–2 years old, five per sex) were used for various assays in this study, with three additional dogs used for examining wagging asymmetry for five consecutive days. The dogs were housed in pairs after weaning at postnatal day 50 in 2 m × 0.9 m × 1.5 m (length × width × height) cages and maintained on a 12 h/12 h dark light cycle, with a humidity of 40%–60% and a temperature of 22–24°C. The dogs were fed with Royal Canine Chow (Royal Pet Food Company Ltd., France) twice daily from 08:00–10:00 and 15:30–17:00. Based on a veterinary assessment, all dogs were in good health at the time of the experiments. All tests were performed at similar times of day (9:00–12:00 or 14:30–17:00) and after food delivery. No dogs were in estrus during the experiments.

For individual animals, behavioral recordings were performed once daily in 5-min sessions for three or five consecutive days. Before the recording, each dog was guided into the recording arena (2 m × 2 m) surrounded by fences, and allowed to move freely for 10 min. An experimenter then approached the fence and interacted with the animal for 5 min, including feeding the dog with snacks approximately every minute, while walking around the fences. The same experimenter conducted all social interactions with dogs.

### Method details

#### 3D markerless motion-tracking setup

We performed motion-capture recordings using a five-camera motion-capture system. Specifically, five cameras (MV-CA016-10UC, Hikvision, China; resolution 720 × 540 at up to 150 frames/s) with varifocal lenses (L2004-5M) were set up on tripods above and around the sides of the recording arena. All cameras were positioned 1 m from the center of the recording arena at two different heights and angled at 90° (center camera) or 70° (cameras near four corners) to the ground, to track the key body parts of the dogs (Figure 1A). The videos were recorded using commercial software (Machine Vision Software MVS 2.0.0, Hikrobot) operating on a computer (with 16 GB RAM and 3.6 GHz Intel i7 CPU). We used an external synchronization TTL pulse (12 V) as a trigger to synchronize all cameras using General Purpose Input/Output. The pulses were generated by a high-precision waveform generator, with a 70 ns rising and falling time for each pulse.

Geometric camera calibration and estimation of the parameters of each camera were performed before 3D pose reconstruction. Parameters were used to correct for lens distortions and to determine the locations of the cameras within the scene. We used a carpet (1.8 m × 1.8 m) with non-repeating visual patterns to facilitate matching of visual features across different viewing angles. A standard structure-from-motion algorithm was used to reconstruct the carpet and five camera poses, including intrinsic and extrinsic parameters, automatically (Schonberger and Frahm, 2016).

#### 3D markerless motion-tracking

We performed preprocessing and data analysis on a computer (with 32 GB RAM, and 3.8 GHz Intel i7 CPU) using MATLAB (Mathworks, Natick, MA, USA) and Python. To quantify the movement trajectories, we tracked the 3D positions of the withers, back, croup, and tail tip frame-by-frame during the entire 5-min recording sessions for 3 days. To this end, we first tracked the 2D position in each video and then reconstructed the 3D position. 2D markerless body-part tracking was achieved using DLC, based on supervised deep neural network (101-layer ResNet) to track visual features in different frames of a video (Mathis et al., 2018). Using tools provided by DLC, we labeled 3000 frames evenly selected from all 30 sessions (three sessions/animal), with 300 frames from each animal. The accuracy of DLC was assessed by cross validation using 80% of the labeled data as the training set and 20% as the testing set. The network provided a likelihood estimate for each of the four body parts. To ensure accurate tracking, we removed all results with a likelihood < 0.9. For the estimations included in further analyses, the root mean squared error was 3.63 pixels for the testing set. The 2D key points were then triangulated into 3D. To obtain the correspondence between 2D tracking points on images and 3D tracking points (the projection matrix), we applied the algorithm of direct linear transform with camera intrinsic and extrinsic parameters obtained from the camera calibration process (Hartley and Zisserman, 2003). The 3D reconstruction was carried out by a custom Python script similar to that used in DLC (Nath et al., 2019).

The 3D data of the key points were smoothed using a Gaussian filter to reduce the effect of noise in the estimation (Wiltschko et al., 2015). For analysis of tail wagging, we aligned all the markers of the animal’s spine and placed markers of the croup at the origin. All the spine markers were then oriented along the *y* axis.

To remove video segments without wagging, we set a sliding time window of 0.3 s (50 frames) and obtained the standard deviations for these segments. Segments with a standard deviation of tail tip coordinates < 1.2 cm were excluded.

#### Kinematic parameters for characterizing tail wagging

To obtain the tail wagging angle, the positions of all markers in 3 dimensions were projected onto the ground plane, which was constructed by positions of the corners. The projection of tail tip on the ground plane connecting the croup and the animal’s spine formed an angle. The angle of the tail on the left side denoted as a negative value, while the angle of the tail on the right side denoted as a positive value.

We defined the extrema of tail wagging angle along time as the angle of wagging. The velocity of waging was defined as: $v=\frac{\theta_{i}-\theta_{j}}{t_{i}-t_{j}}$ ($\theta_{i}$ and $\theta_{j}$: the adjacent angle of wagging; $t_{i}$ and $t_{j}$: the timestamp of tail wagging angle $\theta_{i}$ and $\theta_{j}$, respectively) (Figure 1E).

The amplitude of wagging was defined as: $\left|\theta_{i}-\theta_{j}\right|$ (Figure 1E).

#### Asymmetric tail wagging analysis

The asymmetric tail wagging index was defined as: $\frac{\left(X_{l}-X_{r}\right)}{max\left(\left|X_{l}-X_{r}\right|\right)}$, where $X_{l}$ represents the counts of dots whose angle of wagging are negative (left), and $X_{r}$ represents the counts of dots whose angle of wagging are positive (right).

Numeric data of all animals in the joint distributions (Figures 1F) was grouped into 100 × 100 2-D bins in the bivariate histograms. In each row of bivariate histograms, $X_{l}-X_{r}$ was calculated. All 10 animals’ data formed a 30 × 100 matrix with the asymmetric tail wagging index calculated for each row (Figure 2B).

The number of frames in which the tail wagging to the left and right side was counted for three consecutive days. In each day, the 5-minute recording session was divided into 15 segments, with each one further divided into 30 sub-segments. The number of segments in which the number of frames containing the tail wagging to the left side are different from that to the right side with statistical significance (p < 0.05) by Welch’s *t*-test was counted each day. All 10 animals’ data were analyzed and the number of left-wagging segments, right-wagging segments and no-bias segments were compared for three consecutive days.

#### Clustering method based on dynamic time warping and affinity propagation

The tail wagging angle was used for bout segmentation. The tail wagging from the left to the right then back to the left defines a bout. In total, we obtained 20674 wagging bouts from all recordings. These individual wagging bouts were analyzed with a pipeline involving three steps. First, we computed the distance between each pair of bouts by dynamic time warping (DTW) (Shokoohi-Yekta et al., 2017). Specifically, let X = ($x_{0},\;x_{1},\;\ldots ,\;x_{i},\;\ldots ,\;x_{n}$) and Y = ($y_{0},\;y_{1},\;\ldots ,\;y_{j},\;\ldots ,\;y_{m}$) represent two segments of tail wagging lasted for *n* and *m* time steps, respectively. DTW was computed as the Euclidean distance between aligned segments:

(Equation 1) $$DTW\left(X,Y\right)=min\left(\sqrt{{\sum_{(i,j)\in \pi }\left\|X_{i}-Y_{j}\right\|}^{2}}\right)$$

where *π* was the set of all admissible paths. In these paths, each was a sequence $\left[\pi_{0},\;\ldots \;,\;\pi_{k-1}\right]$ of index pairs $\pi_{k}$ = ($i_{k},\;j_{k}$) with $0\;\leq \;i_{k}\;<\;n$ and $0\;\leq \;j_{k}\;<\;m$, $\pi_{0}=\left(0,0\right)$ and $\pi_{k-1}=\left(i_{k-1},\;j_{k-1}\right)$. For all *k* > 0, $\pi_{k}$ = ($i_{k},\;j_{k}$) was related to $\pi_{k-1}$ = ($i_{k-1},\;j_{k-1}$) as follows:

$i_{k-1}\;\leq \;i_{k}\;\leq \;i_{k-1}+1$, $j_{k-1}\;\leq \;j_{k}\;\leq \;j_{k-1}+1$. $X_{i}$ and $Y_{i}$ were the i^th^ and j^th^ tail wagging segments, respectively.

Second, we combined similar bouts into clusters by affinity propagation clustering (Frey and Dueck, 2007). Finally, we used cluster ID distribution in individual sessions as the feature, and calculated cosine distances between pairs of sessions, which produced a distance matrix that could be hierarchically clustered (Sorensen, 1948) as shown in Figure 3D.

#### Computing the silhouette score of each wagging bout

The silhouette score is calculated using the mean intra-cluster distance of the *i*^th^ wagging bout ($a_{i}$) and the mean nearest-cluster distance of the *i*^th^ wagging bout ($b_{i}$). The silhouette score for the *i*^th^ wagging bout is $\frac{b_{i}-a_{i}}{max\left(a_{i},\;b_{i}\right)}$ (Rousseeuw, 1987).

#### Lyapunov stability analysis of tail wagging

The Lyapunov exponent provides a measure of the stability of a dynamical system and can be calculated from an observable output (in this case, the tail wagging trajectory). Mathematically, it measures the average uncertainty along the local eigenvectors of an attractor in the state space. The largest Lyapunov exponent (LLE) was calculated by using the algorithm proposed by Wolf et al. (Wolf et al., 1985). Specifically, given the time series $\left\{\begin{array}{cc} x_{1}, & x_{2}, \end{array}\;\begin{array}{cc} \ldots & ,\;x_{n} \end{array}\right\}$, an *m*-dimensional phase space was reconstructed with *τ* delay coordinates, which was described as:

(Equation 2) $$Y\left(t_{i}\right)=(x\left(t_{i}\right),\;x\left(t_{i}+\tau \right),\ldots ,\;x\left(t_{i}+\left(m-1\right)\tau \right)),\;i=1,2,\ldots ,n-\left(m-1\right)\tau$$

We defined the initial point in this phase space as $Y\left(t_{0}\right)$ and the nearest neighbor to this initial point as $Y_{0}\left(t_{0}\right)$. The distance between these two points was defined as $L\left(t_{0}\right)=\left|Y\left(t_{0}\right)-Y_{0}\left(t_{0}\right)\right|$. These two points were tracked along the time until the distance of two points at time $t_{1}$, which was defined as

(Equation 3) $$L'\left(t_{1}\right)=\left|Y\left(t_{1}\right)-Y_{0}\left(t_{1}\right)\right|>\varepsilon ,\;\varepsilon >0$$

was larger than ε. Then the point $Y\left(t_{1}\right)$ was retained, and the neighbor to $Y\left(t_{1}\right)$ was defined as $Y_{1}\left(t_{1}\right)$, which satisfied two criteria: the distance of these two points $L\left(t_{1}\right)=\left|Y\left(t_{1}\right)-Y_{1}\left(t_{1}\right)\right|$ was less than ε and the angle $\theta_{1}$ between the evolved vector $L'\left(t_{1}\right)$ and replacement vector $L\left(t_{1}\right)$ was minimal among all replacement vectors $L\left(t_{1}\right)$ that met the first criterion. This procedure was repeated until the end of the time series. We estimated the largest Lyapunov exponent (LLE) λ  as follows:

(Equation 4) $$\text{λ}=\frac{1}{M}\sum_{k=1}^{M}\log{\;}_{2}\frac{L'\left(t_{k}\right)}{L\left(t_{k-1}\right)}$$

where *M* was the total number of replacement steps (Wolf et al., 1985). We set a time window of 2 s (300 frames), which slid through the tail wagging datasets with padding of 10 frames. We embedded these segments using an embedding dimension *m* = 3, a time delay τ = 100, the maximum separation at replacement *ε* = 0.16. Three largest Lyapunov exponent (LLE) values along the *x-*, *y-* and *z*-axis were obtained for each 300 frames segment. The sum of three LLE values for each segment indicates the stability of tail wagging. For visualizing the temporal evolution of LLE in Figure 4A, the curve was smoothed by the Gaussian-weighted moving average filter. An LLE threshold of 0.18 (the 25^th^ percentile of LLE distribution, which ranging from −0.01 to 0.98) was set to divide the data into two categories: stable states (LLE < 0.18) and unstable/transient states (LLE > 0.18).

#### Lyapunov stability-based approach to segment tail wagging modules

We applied a Lyapunov stability-based approach to segment stable states of tail wagging. Tail wagging varies in frequency, so a sliding window with fixed size mentioned above is not suitable for segmentation. To address this, we used wagging bouts as the basic units in segmentation. First, we split the tail tip trajectory with a spatial constraint, and repeated the calculation as follows: $d=\sqrt{\left(a-b\right){\left(a-b\right)}^{T}}$ (a and b represent the start and end of the consecutive bouts, respectively) until the Euclidean distance d was larger than 0.8 cm, and the consecutive bouts were retained and seen as a segment. Second, the segments were further divided into stable segments and transient segments (Figures 4A and 4B). Third, we computed the distance between each pair of segments by dynamic time warping (DTW), which handles temporal offsets and small variations better than Euclidean distance (Sakoe and Chiba, 1978). Similar segments were combined by affinity propagation clustering (Frey and Dueck, 2007), which generated micro-modules of wagging patterns (Figure 4C). The prototypical segments that are representative of these micro-modules, were combined into mini-modules by affinity propagation clustering. DTW is sensitive to noise because its basic unit is to compute point-wise Euclidean distance between points and it relies heavily on the sequence shape during alignment (Gong and Chen, 2018). To address this issue, we embedded each exemplar of the clusters into a 100 × 100 × 100 tensor, and dimension reduction was performed by principal component analysis (Pearson, 1901). At the final step, the extracted data features from dimension reduction were processed by *k*-means clustering (Lloyd and Johnson, 1982), which yielded wagging modules.

#### Algorithm for visualizing the joint distribution

The joint distribution was visualized by using heat maps. Specifically, a sample data point ($a_{i},b_{i}$) on the 2D X-Y plane of distribution was transformed into the *i*^th^ 2D Gaussian kernel centered at ($a_{i},b_{i}$), which can be calculated by the following equation:

(Equation 5) $$Z_{i}=Se^{\frac{-\left({\left(X-a_{i}\right)}^{2}+{\left(Y-b_{i}\right)}^{2}\right)}{\sigma^{2}}}$$

where $a_{i}$ is the *i*^th^ angle of tail wagging along a 5-min session in Figures 1F and 1G, and $b_{i}$ is either the *i*^th^ amplitude of wagging (Figure 1F) or the *i*^th^ velocity of wagging (Figure 1F) along a 5-min session. The variance *σ* and the scaling factor *S* were both set to be 0.5. X is a matrix with vectors [-A, $\begin{array}{lll} -A+c, & \ldots & ,\;-A+\left(n-1\right)c \end{array}$ ,-A+nc] of 400 repeated rows. Y is a matrix with vectors [0, $\begin{array}{lll} d, & \ldots & ,\;\left(m-1\right)d,\; \end{array}$ md] of 400 repeated rows (*n* = 400, *m* = 400, *c* = 1; for Figure 1F, *d* = 0.9; for Figure 1G, *d* = 0.1).

The heat map matrix *H* is calculated as follows:

*H*= $1-\left(1-Z_{1}\right)\circ \left(1-Z_{2}\right)\cdots \left(1-Z_{n}\right)$, where ∘ is element-wise production. Finally, the elements of heat map matrix *H* are color-coded for visualization.

### Quantification and statistical analysis

Statistical analysis was performed using GraphPad Prism and MATLAB. Differences between the numbers of frames with tail wagging to the left and right side were analyzed by two-tailed Welch’s *t*-test. The significance of differences in numbers of left-wagging segments, right-wagging segments, and no-bias segments on different days was determined by Mann-Whitney test. Significance was set to be p < 0.05, adjusted for multiple comparisons by the Holm-Šídák method.

## Data Availability

• The accession URL for the datasets is listed in the key resources table. • The code generated during this study and sample data are available on Mendeley Data: https://doi.org/10.17632/sfp7k9hzkx.1 • Any additional information required to reanalyze the data reported in this paper is available from the lead contact upon request.
