# Supplementary material for: Left-right asymmetry and attractor-like dynamics of dog’s tail wagging during dog-human interactions
Source: iScience. 2022 Jul 9;25(8):104747. doi: 10.1016/j.isci.2022.104747 (PMC9356099; doi:10.1016/j.isci.2022.104747)
Supplement: Document S1. Figures S1 and S2 [file mmc1.pdf]

iScience, Volume *25*

Supplemental information

Left-right asymmetry and attractor-like dynamics of dog's tail wagging during dog-human interactions

Wei Ren, Pengfei Wei, Shan Yu, and Yong Q. Zhang

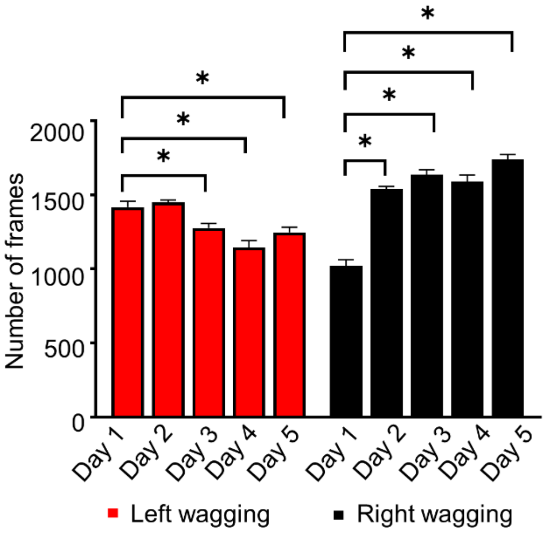

Figure S1: Tail wagging shifts from left- to right-side bias for additional three animals across five days. Related to Figure 2. The numbers of frames in which the tail wagging to the left or right side (color-coded) are plotted for five consecutive days. Error bars represent the standard error of the mean (SEM). ns, no significance; *p < 0.05 by Welch's *t*-test.

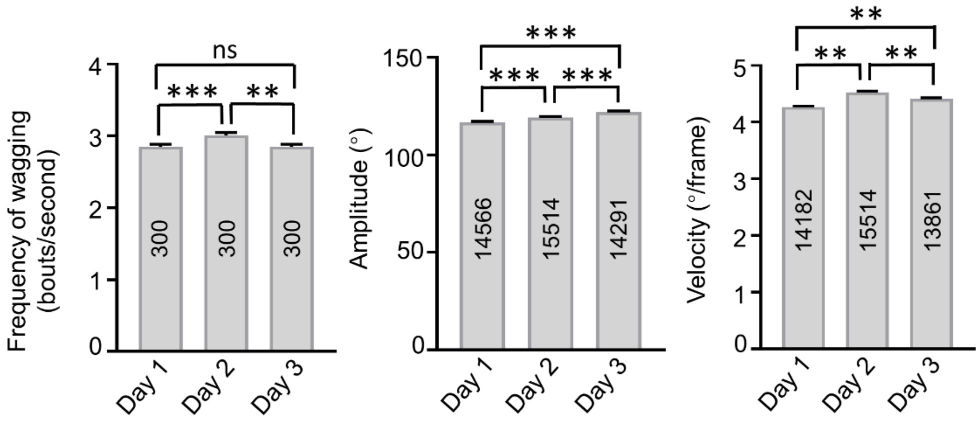

Figure S2: Comparisons of the frequency, amplitude, and velocity of wagging for ten animals across three days. Related to Figure 2. The results of frequency (left panel), amplitude (middle panel), and velocity of wagging (right panel) for ten animals are plotted for three consecutive days. Error bars represent SEM. The sample size was noted in the bar. ns, p >0.05; **p < 0.01; ***p < 0.001 by Mann-Whitney test.
